# Fibroblasts from patients with Diamond-Blackfan anaemia show abnormal expression of genes involved in protein synthesis, amino acid metabolism and cancer

**DOI:** 10.1186/1471-2164-10-442

**Published:** 2009-09-18

**Authors:** Federica Avondo, Paola Roncaglia, Nicoletta Crescenzio, Helena Krmac, Emanuela Garelli, Marta Armiraglio, Carlotta Castagnoli, Maria Francesca Campagnoli, Ugo Ramenghi, Stefano Gustincich, Claudio Santoro, Irma Dianzani

**Affiliations:** 1 Department of Medical Sciences and Interdisciplinary Research Center of Autoimmune Diseases (IRCAD), Università del Piemonte Orientale, Novara, Italy; 2 International School for Advanced Studies (SISSA/ISAS), Trieste, Italy; 3 Department of Pediatric Sciences, Università di Torino, Torino, Italy; 4 Azienda Ospedaliera CTO/CRF/Maria Adelaide, Torino, Italy

## Abstract

**Background:**

Diamond-Blackfan anaemia (DBA) is a rare inherited red cell hypoplasia characterised by a defect in the maturation of erythroid progenitors and in some cases associated with malformations. Patients have an increased risk of solid tumors. Mutations have been found in several ribosomal protein (RP) genes, i.e *RPS19, RPS24, RPS17, RPL5, RPL11, RPL35A*. Studies in haematopoietic progenitors from patients show that haplo-insufficiency of an RP impairs rRNA processing and ribosome biogenesis. DBA lymphocytes show reduced protein synthesis and fibroblasts display abnormal rRNA processing and impaired proliferation.

**Results:**

To evaluate the involvement of non-haematopoietic tissues in DBA, we have analysed global gene expression in fibroblasts from DBA patients compared to healthy controls. Microarray expression profiling using Affymetrix GeneChip Human Genome U133A 2.0 Arrays revealed that 421 genes are differentially expressed in DBA patient fibroblasts. These genes include a large cluster of ribosomal proteins and factors involved in protein synthesis and amino acid metabolism, as well as genes associated to cell death, cancer and tissue development.

**Conclusion:**

This analysis reports for the first time an abnormal gene expression profile in a non-haematopoietic cell type in DBA. These data support the hypothesis that DBA may be due to a defect in general or specific protein synthesis.

## Background

Protein synthesis is essential to the survival and growth of cells. Ribosomes, the sites where translation occurs, therefore play a fundamental role in cell biology. In human cells, ribosome biogenesis occurs in the nucleolus: it requires the transcription of four ribosomal RNA (rRNA) species and their assembly with 79 ribosomal proteins (RPs) in order to produce the small (40S) and large (60S) ribosomal subunits. These subunits are independently exported to the cytoplasm and joined to obtain mature ribosomes [1].

Several inherited or acquired bone marrow failure syndromes are due to mutations in genes encoding proteins involved in ribosome biogenesis. They include Diamond-Blackfan anaemia (DBA, MIM#105650), Shwachman-Diamond syndrome (SDS, MIM#260400), dyskeratosis congenita (DC, MIM#127550, #305000, #224230) and 5q- syndrome (MIM#153550) [2,3]. DBA is an inherited erythroid hypoplasia which usually develops within the first year of life and is characterised by a severe normochromic macrocytic anaemia caused by a defect in the maturation of erythroid progenitors. Haematological signs include paucity of bone marrow progenitors, reticulocytopenia, elevated erythrocyte adenosine deaminase (eADA) activity and high levels of foetal haemoglobin. Patients are prone to develop malignancies and one third of them present congenital abnormalities [4,5]. Mutations causing DBA have been found so far in six RP genes, encoding both small and large subunit components: *RPS19 *(in 25% of cases), *RPS24 *(2%), *RPS17 *(one case), *RPL35A *(2%), *RPL5 *(9%) and *RPL11 *(6.5%), overall accounting for about 50% of cases [6-10]. All patients are heterozygous with respect to mutations and haplo-insufficiency is thought to be responsible for the pathogenetic mechanism of the disease. The main hypothesis to explain DBA implies that a defect in ribosome biogenesis and protein synthesis would trigger apoptotic processes in erythroid progenitors. This is supported by several studies showing that mutations in RPs impair rRNA processing, both in CD34^+ ^cells from DBA patients and in erythroid cells with knock-down of the known DBA genes [8-13]. Mutations in different genes impair different steps of rRNA maturation, but they all lead to the accumulation of rRNA precursors and to a reduction in mature ribosomes. Similar alterations have also been demonstrated in yeast and human cells deficient for other RPs, not yet found mutated in DBA [2,14,15]. However, the link between the haplo-insufficiency of an RP and the erythroid defect occurring in DBA has not been clarified yet.

DBA has been considered a disease that affects only erythroid progenitors and thus the prototype of a pure erythroid aplasia. However, several lines of evidence suggest that erythroid progenitors, though apparently more sensitive to RP haplo-insufficiency than other cell types, are not the only cell type affected in DBA. Some patients evolve trilinear aplasia [16], showing that haematopoiesis in general can be affected, while the presence of congenital malformations demonstrates that some organogenetic processes are affected by DBA mutations. Moreover, patients show short statures well below their genetic potentials [5].

To gain a better insight into the biological processes and functions involved in the pathogenesis of DBA, we performed a global gene expression analysis of fibroblasts isolated from DBA patients carrying mutations in *RPS19*. Our data reveal for the first time the presence of abnormal gene expression in non-haematopoietic DBA cells. Protein synthesis, nucleotide and amino acid metabolism and apoptosis are the most affected biological processes.

## Results

### Gene expression profiling of cells from DBA patients

To identify genes associated with a defect of RPS19, a whole genome expression profiling study was performed using Affymetrix GeneChip Human Genome U133A 2.0 Arrays which allow the screening of 18,400 transcripts, including 14,500 well-characterised genes. We analysed the gene expression profiles of dermal fibroblasts isolated from four DBA patients carrying mutations in *RPS19*, in comparison to those obtained from six healthy individuals. Of the 22,227 probes present on the chip, 13,396 had Affymetrix "Present" detection calls and intensity values more than 100 in all arrays and have been used for data analysis. A list of 490 statistically significantly differentially expressed probes was generated, 215 of which are up-regulated and 275 down-regulated in DBA patients (see additional file 1: Genes differentially expressed in DBA patients identified by microarray profiling). These probes correspond to 421 differentially expressed genes (176 up-regulated and 245 down-regulated) in patients compared to controls.

We also evaluated whether the observed differences in gene expression may be due to a gender-dependent bias. The comparison between the array data of female to male individuals irrespective of the health status yielded a list of 33 differentially expressed probes (six up-regulated and 27 down-regulated in females), corresponding to genes mainly located on chromosomes X or Y (see additional file 2: Differentially expressed genes in females relative to males). Moreover, we identified only five differentially expressed genes in female DBA patients relative to male DBA patients (see additional file 3: Differentially expressed genes in female patients relative to male patients). These results rule out the presence of gender-dependent defects in DBA patients.

Microarray analysis was performed on samples from patients carrying two missense (p.Arg62Trp and p.Arg101His), one frameshift and one splice site mutations (c.58delG and c.1-1G>A) in *RPS19 *(table 1). We did not identify any gene showing a statistically significant differential expression in patients with missense mutations compared to other mutation types, indicating that changes due to the specific nature of the mutational status were not found in our study.

**Table 1:** Clinical characteristics of DBA patients

**Patient**	**Gender**	**Mutation ** **(RPS19)**	**Steroid ** **Response**	**Follow-up**	**Congenital ** **Abnormalities**
1	M	p.Arg62Trp	R/I^a^	BMT/Rem^e^	yes
2	F	p.Arg101His	R^b^	TRT/Rem^f^	no
3	F	c1-1G>A	NR^c, d^	BMT/Rem	no
4	M	c.del58G	R	SD^g^	no

^a ^R/I responsive and subsequent interruption of therapy

^b ^R responsive

^c ^this patient was transfusion dependent

^d ^NR non responsive

^e ^BMT/Rem remission after bone marrow transplant

^f ^TRT/Rem remission after treatment

^g ^SD steroid dependent

**Figure 1:**
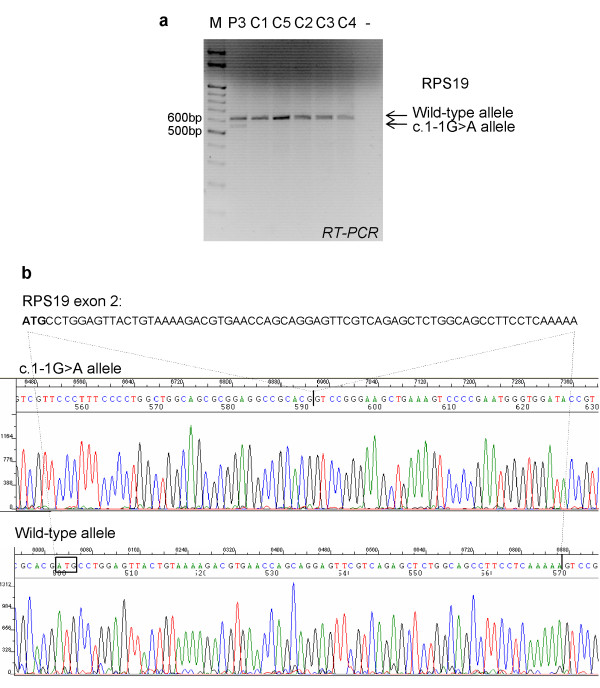
**RPS19 expression in patient 3**. a) RPS19 expression in patient 3 and controls is shown. Primers complementary to exon 1 (forward) and exon 4 (reverse) were used. Two PCR products are indicated in lane 1 at 628 and 557 bp. b) Sequencing of *RPS19 *in patient 3. Exon 2 is 71 nt long and the first three nucleotides represent the translation initiation start site (bold). The chromatogram shows deletion of exon 2 in c.1-1G>A allele compared to the wild-type sequence in patient 3.

Microarray analysis did not show any statistically significant difference in the mean expression of *RPS19 *when the group of patients is compared to the group of controls. This is due to the fact that two patients carried missense mutations that are expressed similarly to controls. Inspection of the microarray data relative to each patient showed that patient 4 with the frameshift mutation c.del58G had reduced *RPS19 *levels (fold change 0.68) as expected by activation of NMD [17,18]. A slightly reduced *RPS19 *expression was found also in patient 3 carrying mutation c.1-1G>A (fold change 0.84). This was confirmed by qRT-PCR with a fold change of 0.76 as compared to five controls (data not shown). Mutation c.1-1G>A affects intron 1 acceptor splice site and is expected to cause exon 2 skipping. PCR using primers complementary to exon 1 (forward) and exon 4 (reverse) revealed the presence of two amplicons of 628 and 557 bp in patient 3 (figure 1a). Sequencing of PCR products confirmed that the smaller abnormal transcript lacks exon 2 which contains the canonical ATG (figure 1b). The first available ATG at position 96-98 in exon 3 is out-of-frame (nucleotide numbering uses the A of the ATG translation initiation start site as nucleotide 1): this transcript is expected to be degraded by NMD [17,18]. However, we cannot rule out that the use of the in-frame ATG at codon 75 in exon 4 would generate a short protein of 71 aminoacids. Either way, a loss of function effect is expected to occur by this mutation in agreement with the assumed haploinsufficiency theory of DBA pathogenesis.

#### Biological processes altered in DBA patients

In order to systematically detect impaired biological processes and molecular functions, we analysed the dataset of genes differentially expressed in patients relative to controls through the use of Ingenuity Pathways Analysis [19]. The analysis identified a statistically significant enrichment of genes that belong to the following pathways (table 2): aminoacyl-tRNA biosynthesis (*LARS, WARS, GARS, SARS, QARS, EPRS*), glycine, serine and threonine metabolism (*PSAT1, PHGDH, GARS, CTH, SARS, SHMT2*), death receptor signaling (*CASP9, NFKBIA, NFKBIE, APAF1, NFKB2, NFKB1, BIRC3*), role of PKR in interferon induction and antiviral response (*CASP9, NFKBIA, NFKBIE, APAF1, NFKB2, NFKB1*) and RAR activation (*SRC, PRMT2, RDH11, ADCY3, PRKACA, NFKB2, NFKB1, MAPKAPK2, RBP1, MMP1, PTEN*). In addition, analysis of the molecular function of the annotated genes revealed enrichment of gene clusters with roles in protein synthesis, including a large cluster of RP genes (*RPL22, RPL27A, RPL29, RPL31, RPL14, RPL18A, RPS3, RPL18, RPL13, RPL3, RPL34, RPS2, RPS12, RPL15, RPL28*), cell death, cellular development, lipid metabolism and molecular transport. Additionally, we identified clusters of genes belonging to the "Top Bio Functions" category of molecular and cellular functions (table 3) and diseases and disorders, which comprises cancer related genes, as well as genes involved in haematological, immunological, renal and skeletal disorders (table 4). Finally, we observed enriched clusters of genes related to the development and function of embryonic, skeletal and connective tissues (table 5).

**Table 2:** Ingenuity Pathways Analysis: Top canonical pathways.

**Pathway**	-Log(P-value*)	Ratio**	**Molecules**	**N°**
Aminoacyl-tRNA Biosynthesis	3.36E+00	7.14E-02	LARS, WARS, GARS, SARS, QARS, EPRS	6
				
Role of PKR in Interferon Induction and Antiviral Response	2.84E+00	1.28E-01	CASP9, NFKBIA, NFKBIE (includes EG:4794), APAF1, NFKB2, NFKB1	6
				
Death Receptor Signaling	2.73E+00	1.08E-01	CASP9, NFKBIA, NFKBIE (includes EG:4794), APAF1, NFKB2, NFKB1, BIRC3	7
				
Glycine, Serine and Threonine Metabolism	2.31E+00	4.17E-02	PSAT1, PHGDH, GARS, CTH, SARS, SHMT2	6
				
RAR Activation	2.28E+00	6.04E-02	SRC, PRMT2, RDH11, ADCY3, PRKACA, NFKB2, NFKB1, MAPKAPK2, RBP1, MMP1 (includes EG:4312), PTEN	11

*Fischer's exact test was used to calculate a p-value determining the probability that each canonical pathway assigned to that data set is due to chance alone.

**Number of genes from the dataset that map to the pathway divided by the total number of molecules that exist in the canonical pathway

**Table 3:** Ingenuity Pathways Analysis: Top Bio Functions - Molecular and Cellular Functions

**Category**	P-value*	**Molecules**	**N°**
Protein Synthesis	3.67E-07 to 3.06E-02	MME, RPL22, UBE2H (includes EG:7328), RPL27A, LIF, RPL29, MMP14, RPL31, RPL14, IDE, NFKB1, EIF4EBP1, CASP9, TRHDE, RPL18A, EDN1, CTSS, MRP63, EIF5, RPS3, RPL18, MMP1 (includes EG:4312), RPL13, SRC, RRBP1, PJA1, EIF3H, RPL3, RPL34, C9ORF3, RPS2, APAF1, CEBPB, DCTN2, RPS12, KIAA0368, RPL15, WARS, RPL28, USE1, SGSM3, IFT52, SMURF2, PLAU	44
			
Cell Death	1.78E-04 to 3.06E-02	LIF, ATXN1, NME2, CDKN2C, TMEM132A, ARG2, MSX2, RBP1, PTEN, SOD2, CASP9, CCNB1IP1, CTSS, PQBP1, SUB1, NUPR1, FOSL2, BIRC3, HLA-C, FBL, NOX4, RP5-886K2.1, NOVA1, YWHAE, SGCG, MITF, FGFR1, THRA, JUNB, NFKB2, NDN, ADI1, OGG1, CCND2, RAPGEF2, BTG2, PRKACA, RUNX1, CYLD, LRIG1, MME, BRD2, GABPB2, ICAM1, RGS3, MMP14, KLF10, BMP2, SAT1, EXT1, DUSP22, TNFAIP3, FKBP1A, FNTA, NFKB1, QARS, ASNS, RPLP0 (includes EG:6175), COMP, EIF4EBP1, PGF, RASSF1, ANGPTL4, NFKBIA, EDN1, FOXO3, ACTC1, MMP1 (includes EG:4312), ATN1, B4GALT5, MUC1, CALR, SRC, PXN, PRMT2, THG1L, PPIF, BGN, ELL, APAF1, PPP1R15A (includes EG:23645), CEBPB, CLCF1, IGF2R, PRG2 (includes EG:5553), NUP62, MAFB, CTH, PLAU, ATP2B4	90
			
Cellular Development	2.14E-04 to 3.06E-02	LIF, NME2, SFPQ, MSX2, EEF1D, LIMK1, PTEN, SERPINB2, NR4A3, CDON, POSTN, NUPR1, FOSL2, NOX4, NAB1, MITF, FGFR1, THRA, L1CAM, NFKB2, MBD2, JUNB, NDN, CCND2, BTG2, RUNX1, MAST2, MSC, ICAM1, NLGN1, MMP14, BMP2, KLF10, MCC, METTL8, TNFAIP3, EXT1, NFKB1, PGF, RASSF1, NFKBIA, EDN1, FOXO3, ATN1, MMP1 (includes EG:4312), SRC, NHEJ1, CALR, PXN, RELB, ELL, APAF1, IRAK3, CEBPB, IGF2R, CLCF1, AFF1, RBPJ, MAFB, PLAU	60
			
Lipid Metabolism	2.41E-04 to 3.06E-02	MUC1, SRC, CASP9, EDN1, APAF1, PHGDH, FAR2, ABCA1, PTEN	9
			
Molecular Transport	2.41E-04 to 3.06E-02	CACNA1G, CALR, SRC, YWHAE, ATXN1, NUP133, SAT1, APAF1, NUP50, ABCA1, CASP9, EDN1, SMG7, PHGDH, ATP2B4	15

*Fischer's exact test was used to calculate a p-value determining the probability that each biological function assigned to that data set is due to chance alone.

**Table 4:** Ingenuity Pathways Analysis: Top Bio Functions - Diseases and Disorders

**Category**	P-value*	**Molecules**	**N°**
Cancer	1.12E-04 to 3.06E-02	RPL22, EPS8, LIF, NME2, SFPQ, CDKN2C, SSH1, PTEN, LIMK1, SERPINB2, SOD2, CASP9, CTSS, POSTN, NEDD4L, BIRC3, EPB41L1, CACNA1G, SOX4, YWHAE, RRAD, FGFR1, L1CAM, MBD2, NFKB2, JUNB, SIP1, ADI1, OGG1, NDN, CCND2, RAPGEF2, BTG2, PRKACA, RUNX1, CYLD, LRIG1, MME, BRD2, ICAM1, GEM, RGS3, BMP2, KLF10, MMP14, MCC, HAX1, SAT1, FKBP1A, EXT1, SEPT9, NFKB1, RPLP0 (includes EG:6175), COMP, EIF4EBP1, PGF, RASSF1, NFKBIA, ANGPTL4, EDN1, FOXO3, TAOK2 (includes EG:9344), MEG3 (includes EG:55384), B4GALT5, MMP1 (includes EG:4312), MUC1, CALR, SRC, PXN, RELB, APAF1, PPP1R15A (includes EG:23645), CEBPB, CLCF1, IGF2R, AFF1, NOV, PLAU	78
			
Hematological Disease	1.12E-04 to 3.06E-02	LIF, ICAM1, MCC, SFPQ, NFKB1, SERPINB2, PTEN, EIF4EBP1, CASP9, NFKBIA, HLA-C, SRC, RELB, ELL, MAN2A1, NFKB2, JUNB, CEBPB, CLCF1, AFF1, CCND2, BTG2, PRKACA, CYLD, PLAU, RUNX1, LRIG1	27
			
Immunological Disease	1.12E-04 to 3.06E-02	ICAM1, LIF, BGN, MMP14, BMP2, RELB, CDKN2C, NFKB2, JUNB, NFKB1, RPLP0 (includes EG:6175), CLCF1, PTEN, SOD2, CASP9, CCND2, NFKBIA, BTG2, FOXO3, RUNX1, CYLD, LRIG1, BIRC3, HLA-C	24
			
Renal and Urological Disease	3.57E-04 to 3.06E-02	SRC, RASSF1, SOD2, EDN1, POSTN, NUPR1, PLAU, LRIG1, SIP1, PTEN	10
			
Skeletal and Muscular Disorders	6.71E-04 to 3.06E-02	MME, LIF, ICAM1, BMP2, MMP14, EXT1, TNFAIP3, C18ORF10, IDE, NFKB1, PTEN, NR4A3, CASP9, SOD2, NFKBIA, EDN1, CTSS, FOXO3, RPS3, MAPKAPK2, MMP1 (includes EG:4312), HLA-C, SRC, NOX4, PPIF, MITF, BGN, APAF1, L1CAM, NFKB2, CEBPB, NDN, DCTN2, CLIC2, NUP62, PLAU, RUNX1, ATP2B4, C20ORF43, MCTP2	40

*Fischer's exact test was used to calculate a p-value determining the probability that each biological function and/or disease assigned to that data set is due to chance alone.

**Table 5:** Ingenuity Pathways Analysis: Top Bio Functions - Physiological System Development and Function

**Category**	P-value*	**Molecules**	**N°**
Connective Tissue Development and Function	2.14E-04 to 3.06E-02	RPL22, ICAM1, LIF, KLF10, BMP2, MMP14, METTL8, HAX1, TNFAIP3, NFKB1, MSX2, EIF4EBP1, PTEN, PGF, LIMK1, EDN1, CHST3, FOSL2, SSBP3, MMP1 (includes EG:4312), SRC, PXN, MITF, BGN, JUNB, IRAK3, CEBPB, NFKB2, IGF2R, NDN, NOV, RUNX1, PLAU	33
			
Embryonic Development	2.14E-04 to 3.06E-02	LIF, ICAM1, BMP2, EXT1, NUP50, MSX2, PTEN, NR4A3, NFKBIA, RASSF1, EDN1, POSTN, FOSL2, SRC, PXN, MITF, FGFR1, THRA, L1CAM, JUNB, CEBPB, AFF1, BTG2, RBPJ, IFT52, PLAU	26
			
Skeletal and Muscular System Development and Function	2.14E-04 to 3.06E-02	MSC, VAMP5, LIF, MMP14, BMP2, KLF10, METTL8, FKBP1A, EXT1, NFKB1, COMP, MSX2, PGF, PTEN, SATB2, NFKBIA, EDN1, CDON, FOXO3, CHST3, POSTN, FOSL2, ACTC1, SSBP3, CACNA1G, SRC, NOX4, BMP2K (includes EG:55589), NAB1, SGCG, MITF, FGFR1, BGN, ADCY3, THRA, NFKB2, IRAK3, JUNB, SMA4, RBPJ, PLAU, RUNX1	42
			
Tissue Morphology	2.14E-04 to 3.06E-02	ICAM1, LIF, BMP2, NFKB1, MSX2, ABCA1, PGF, PTEN, EIF4EBP1, CASP9, NFKBIA, EDN1, CTSS, PHGDH, FOSL2, SSBP3, SRC, RELB, BGN, ZIC1, APAF1, L1CAM, THRA, JUNB, NFKB2, CEBPB, OGG1, AFF1, CCND2, RBPJ, CYLD, PLAU, ATP2B4	33
			
Respiratory System Development and Function	1.44E-03 to 3.06E-02	SRC, NFKBIA, CCND2, COX11, EDN1, MAN2A1, MAFB, ABCA1, NDN, PTEN	10

*Fischer's exact test was used to calculate a p-value determining the probability that each biological function assigned to that data set is due to chance alone.

### Quantitative RT-PCR validation of microarray data

**Figure 2:**
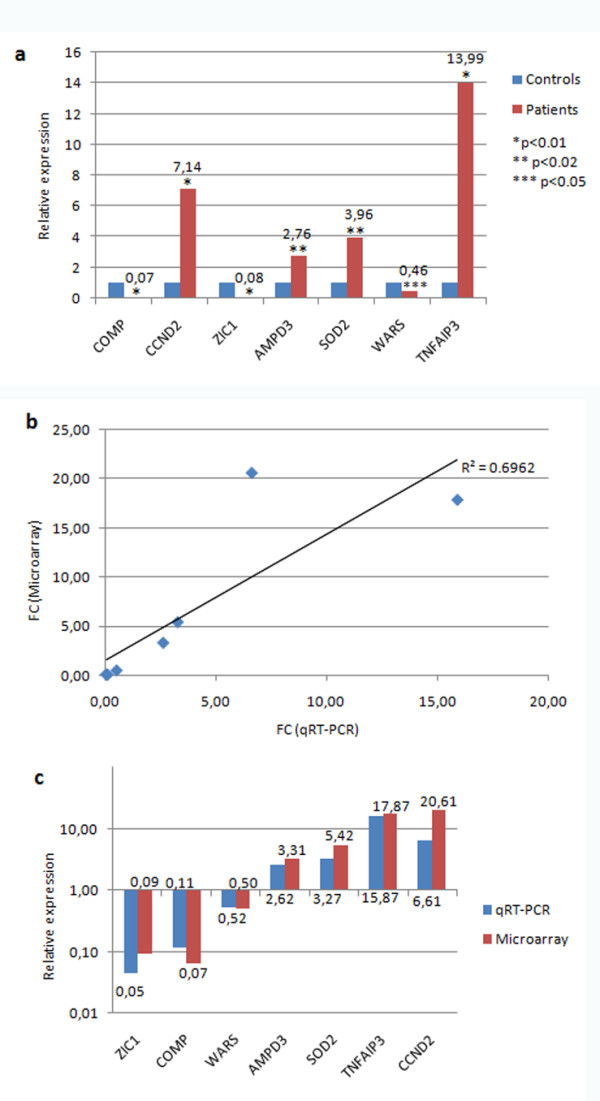
**Gene expression by qRT-PCR**. a) The mean relative expression in the group of DBA patients relative to the group of controls (set equal to 1) is reported for each analysed gene. Beta actin was used to normalise data. P values less than 0.05 were considered as statistically significant. b) Correlation of fold changes in the expression of analysed genes by microarray and qRT-PCR. The R^2 ^coefficient of correlation is reported. c) The mean relative expression in the group of DBA patients relative to the group of controls is reported for each analysed gene. Fold changes obtained from qRT-PCR and microarray analysis are shown for each analysed gene.

**Figure 3:**
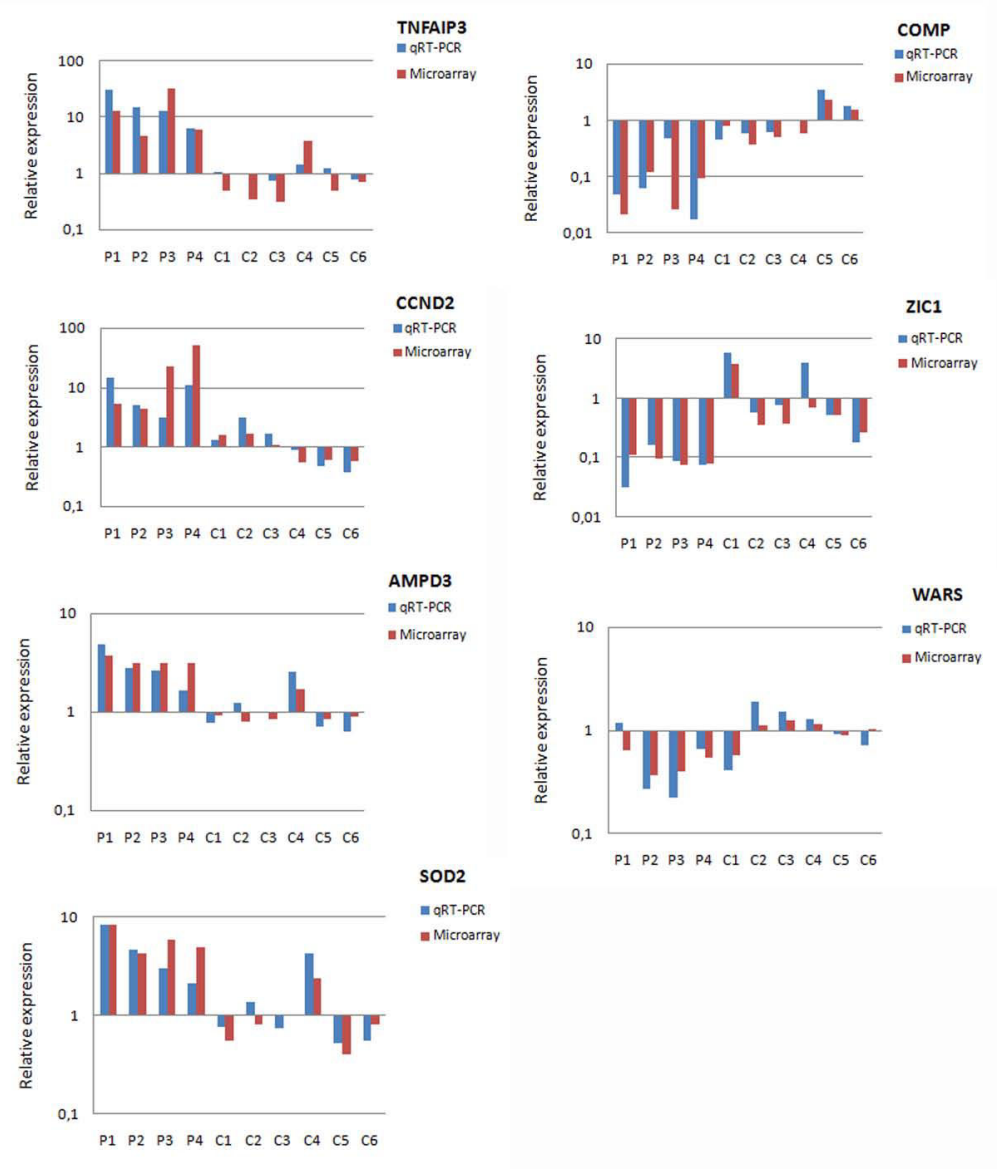
**Comparison between qRT-PCR and microarray fold changes**. The relative expression of genes analysed by qRT-PCR is reported for each sample. The mean expression of controls was used as calibrator (set equal to 1) and beta actin was used to normalise data. Fold changes obtained from microarray analysis are shown for each analysed gene.

In order to corroborate the microarray gene expression results, we selected seven genes among those differentially expressed in DBA patients relative to controls. Real-time RT-PCR was performed on the same RNA samples used for microarray analysis. The expression of *AMPD3*, *CCND2*, *SOD2*, *TNFAIP3 *(up-regulated in DBA patients) and *COMP*, *WARS *and *ZIC1 *(down-regulated in DBA patients) was tested. All genes were found to be differentially expressed in patients relative to controls and data are statistically significant (figure 2a). The correlation between qRT-PCR and microarray data is 0.7 (figure 2b), which corresponds to the value obtained in similar studies [20,21]. Figure 2c shows the comparison of fold changes from qRT-PCR and microarray analysis. Microarray data showed the same trend as qRT-PCR results for all the examined genes in each sample, as represented in figure 3. These data overall indicate that the expression patterns detected by microarray analysis are in good agreement with those detected by qRT-PCR and validate our study.

## Discussion

The most prominent feature in DBA is anaemia due to paucity of erythroid progenitors. However, other cellular types also display molecular alterations. Lymphocytes from patients show reduced proliferation and impaired translational rates irrespective of the nature of the mutation [22]. An abnormal proliferation rate has been found also in fibroblasts from patients with mutations in *RPS19*. Additionally, both lymphocytes and fibroblasts are characterised by impaired pre-rRNA processing with accumulation of 21S species. Fibroblasts show abnormal nucleoli, which have irregular shape and disorganised dense fibrillar centers, the compartments where early pre-ribosome maturation takes place [11].

To characterise the molecular abnormalities in DBA non-haematopoietic cells, we evaluated gene expression profiles of fibroblasts isolated from DBA patients carrying mutations in *RPS19*. Patients 1 and 2 carried mutations leading to amino acid substitutions (p.Arg62Trp and p.Arg101His), the mutation in patient 3 (c.1-1G>A) impairs the correct splicing of the first intron and abrogates the ATG start codon, whereas the mutation in patient 4 (c.del58G) causes a frameshift of the open reading frame with insertion of a premature stop codon. Regardless of the heterogeneity of the mutational types, we did not observe any significant difference when missense cases were compared to the other mutations, although this may be due to the limited number of patient samples of each mutational class in our study.

Gene expression profiling has been previously performed on bone marrow CD34^+ ^cells isolated from three DBA patients with mutations in *RPS19 *and in remission from the disease (i.e. without any treatment for at least 10 years), compared with healthy controls [23], and on CD4^+ ^peripheral blood mononuclear cells from two DBA patients with unknown mutations compared with two acquired aplastic anaemia patients [24]. Our study demonstrates for the first time a global alteration of several biological processes in non-haematopoietic DBA cells. In agreement with previous studies [23,24], we identified a cluster of 22 ribosomal protein genes down-regulated in DBA patients relative to controls. Patients exhibit significant down-regulation of genes encoding proteins important for translation, including several eukaryotic translation initiation factors (*EIF3*, *EIF2*, *EIF4E*), EIF3 and EIF4 interacting proteins (*EIF3S6IP *and *EIF4EBP1*) and the eukaryotic elongation factor 1 δ (*EEF1D*). This may be caused either by the co-regulated transcription of RP genes or by their coordinated post-transcriptional regulation.

Most interestingly, among down-regulated genes we identified a large cluster of aminoacyl-tRNA synthetases (*QARS*, *EPRS*, *SARS*, *GARS*, *LARS*, *WARS*). Aminoacyl-tRNA synthetases (ARS) catalyse the aminoacylation of their cognate tRNAs and thus are key enzymes to maintain the fidelity of protein synthesis. In mammals, additional cofactors, i.e. proteins p18, p38 and p43, and interacting core proteins are required to form a functional multisynthetase complex [25]. The gene encoding p18 (*CDKN2C*) is also down-regulated in DBA fibroblasts. Moreover, ARS contribute to the regulation of amino acid metabolism, which is tightly regulated and essential for ribosome biogenesis and function. Human tryptophanyl-tRNA synthetase (*WARS*) is up-regulated after IFN-gamma treatment, together with indoleamine 2,3-dioxygenase (*IDO*), the enzyme responsible for tryptophan degradation, thus creating a pool of Trp-tRNA and providing a reservoir of Trp available for protein synthesis [26-28]. Interestingly, haem stimulates Trp catabolism enhancing both IDO and WARS enzymatic activities [29,30]. An abnormal haem catabolism has been suggested to occur in DBA [31]. Finally, non canonical functions have been proposed for glutamyl-prolyl-tRNA synthetase (EPRS) in the translational regulation of specific genes containing a GAIT element in the 3'UTR [32]. We demonstrate here that these key regulators are defective in fibroblasts from DBA patients. This suggests that a differential regulation of specific mRNAs may have a role in DBA.

High levels of erythrocyte adenosine deaminase (eADA) activity are a common clinical feature in DBA patients, suggesting that adenine catabolism is stimulated. This most likely happens since DBA cells show the impaired processing of rRNA precursor species, which markedly accumulate in the nucleoli and need to be degraded [14]. Interestingly, we observed an increased expression of adenosine monophosphate deaminase (*AMPD3*), an enzyme of nucleotide break-down involved in the regulation of energetic metabolism in mammalian cells. It catalyses the irreversible deamination of adenylic acid and represents a branchpoint of adenylate nucleotides catabolism, regulating the size of the purine nucleotide pool [33,34]. The maintenance of an appropriate intracellular purine nucleotide concentration range is necessary for cell survival. The increased expression of *AMPD3 *may indicate the need to dispose of an excess purine pool in DBA fibroblasts.

About 4% of DBA patients develop cancer, most frequently acute myeloid leukemia, myelodysplastic syndrome and osteosarcoma [5]. This prevalence is much higher than that in the general population. Thus DBA patients seem to have an increased risk of developing malignancies. Gene expression analysis performed on fibroblasts from DBA patients revealed dysregulation of genes involved in cell death and cancer. The decrease in pro-apoptotic (*CASP9, APAF1*) and oncosuppressor genes (*PTEN*), coupled to the increased expression of some oncogenes (*SRC, CYLD*) and pro-survival genes (*CALR*) may suggest a predisposition for *RPS19 *mutated fibroblasts to carcinogenesis. It is interesting to note that several zebrafish lines carrying heterozygous mutations for RP genes are also prone to develop malignancies [35].

Finally, patient fibroblasts differentially express several genes involved in embryonic and tissue development, including *ZIC1*, strongly down-regulated in patients. In mice, deletion of *zic1 *gene results in cerebellar malformations and axial skeletal abnormalities [36,37]. It is worthwhile stressing that 30-48% of DBA patients display congenital malformations, including abnormalities affecting the skeletal axis, such as preaxial polydactily.

## Conclusion

The global gene expression analysis we performed shed light for the first time on the impaired biological processes in a non-haematopoietic cell type in DBA. We revealed a dysregulation of genes involved in ribosome biogenesis and protein synthesis, as well as amino acid and nucleotide metabolism. These data support the hypothesis that DBA may be due to a defect in general or specific protein synthesis.

## Methods

### Patients and cell culture

This study was performed on four DBA patients, carrying two missense (p.Arg62Trp and p.Arg101His), one frameshift and a splice site mutations (c.58delG and c.1-1G>A), respectively, in *RPS19*. Table 1 reports the main clinical characteristics of these patients. Further information about these mutations and their functional characterisation may be found in [4] and in the DBA genes Database [38,39].

Research was carried out in compliance with the Helsinki Declaration. Dermal biopsies were obtained from the four DBA patients and from six healthy controls after informed consent during surgery for medical reasons that were not connected with this study. Fibroblasts were cultured in IMDM (Iscove's Modified Dulbecco's Medium) supplemented with 4 mM L-glutamine, 10% foetal calf serum, 0.1 mg/ml streptomycin, 100 U/ml penicillin (Sigma-Aldrich) at 37°C and 5% CO_2_. Total RNA was extracted from 10^6 ^cells upon reaching 80% confluence, within 2-5 tissue culture passages.

### RNA isolation, microarray processing and data analysis

Total RNA was isolated using the TRIzol reagent (Invitrogen) according to manufacturer's instructions, and purified with the RNeasy Mini kit (QIAGEN). The quality of total RNA was assessed using an Agilent 2100 Bioanalyzer (Agilent Technologies). RNA was quantified with a NanoDrop 1000 spectrophotometer (Thermo Scientific). A 6 μg-amount of each total RNA sample was labelled according to the standard one-cycle amplification and labelling protocol developed by Affymetrix (Santa Clara, CA). Labelled cRNA was hybridised on Affymetrix GeneChip Human Genome U133A 2.0 Arrays containing over 18,000 transcripts. Hybridized arrays were stained and washed (GeneChip Fluidics Station 450) and then scanned (GeneChip Scanner 3000 7G). Cell intensity values and probe detection calls were computed from the raw array data using the Affymetrix GeneChip Operating Software (GCOS). Further data processing was performed in the R computing environment [40] using packages from the BioConductor software project [41]. Robust Multi-Array Average (RMA) normalisation was applied [42]. Normalised data were then filtered based on the Affymetrix detection call, so that only probes that had a Present call in at least one of the arrays were retained [43]. Probes with low intensity values (less than 100) in all arrays were also excluded from statistical analysis. Data were then imported into the MultiExperiment Viewer (MeV) software [44], and statistical analysis was performed with the SAM (Significance Analysis of Microarrays) module [45], implemented as in [46]. A False Discovery Rate (FDR) of 3% was applied to detect significantly differentially expressed genes in DBA patients versus healthy controls. Functional analysis of differentially expressed genes was performed through the use of Ingenuity Pathways Analysis [19]. Microarray data have been deposited in the NCBI Gene Expression Omnibus (GEO) database with Accession Number GSE14335.

### Validation of data by real time RT-PCR

Genes to be validated were selected on the basis of potential interest and as representative of a wide range of expression fold changes in patients relative to controls. 500 ng of total RNA was reverse transcribed to cDNA using the High Capacity cDNA Archive Kit (Applied Biosystems) and random primers. Quantitative PCR was performed with an AbiPrism7000 instrument (Applied Biosystems) and 1 μl of cDNA was used in a 25-μl final volume reaction containing Power SYBR^® ^Green PCR Master Mix (Applied Biosystems) and specific primers (see additional file 4: Primers used in the expression analysis by qRT-PCR). cDNA from each sample was examined in triplicate in each experiment. Experimental Ct values were normalised to beta actin, used as endogenous control. Gene expression was calculated in each sample relative to the mean of controls, using the formula 2^-ddCt^, where dCt is Ct_gene_-Ct_endo _and ddCt is dCt_sample_- mean dCt_controls_. Differences in gene expression of patients relative to controls were statistically evaluated by *t *test for independent samples.

### Mutation analysis

DBA patient 3 was further analyzed for exon 2 skipping in *RPS19 *transcript. The coding sequence spanning exon 1 and exon 4 was PCR-amplified by standard procedures. PCR products were excised from agarose gel, purified with HiYield Gel/PCR DNA Fragments Extraction Kit (Real Biotech, Banqiao City, Taiwan) and sequenced in both directions using a Big Dye Terminator^® ^v1.1 cycle sequencing kit (Applied Biosystems) and an Abi PRISM^® ^3100 genetic analyzer (Applied Biosystems). Primer sequences are available upon request.

## Authors' contributions

FA performed qRT-PCR analysis, gene clustering analysis, mutation analysis and drafted the manuscript. PR performed bioinformatic and statistical microarray data analysis, participated in the interpretation of the data and critically revised the manuscript. NC and CC isolated and cultured fibroblasts. HK carried out microarray hybridisations. EG and MFC genotyped the patients and carried out RNA isolation. MA participated in gene clustering analysis. UR was involved in diagnosis and follow-up of patients, provided their samples and clinical information. SG, CS and ID contributed to the conception and design of the study, interpretation of the data and critically revised the manuscript for important intellectual content. All authors read and approved the final manuscript.

## Supplementary Material

Additional file 1 **Genes differentially expressed in DBA patients identified by microarray profiling**. The table reports the probeset IDs which are differentially expressed in DBA patients relative to controls, with an FDR of 3%. The gene annotation, chromosome location and fold change of expression in patients relative to controls is also reported. Click here for file

Additional file 2 **Differentially expressed genes in females relative to males**. The table reports the probeset IDs which are differentially expressed in females relative to males, with an FDR of 10%. The gene annotation, chromosome location and fold change of expression in females relative to males is also reported. Click here for file

Additional file 3 **Differentially expressed genes in female patients relative to male patients**. The table reports the probeset IDs which are differentially expressed in female DBA patients relative to male DBA patients, with an FDR of 10%. The gene annotation, chromosome location and fold change of expression in females relative to males is also reported. Click here for file

Additional file 4 **Primers used in the expression analysis by qRT-PCR**. The table reports the sequence of both forward and reverse primers used to amplify genes in the validation experiments by qRT-PCR. The final concentration used in the reactions is also indicated for each primer. Click here for file
