# Supplementary material for: Fibroblasts from patients with Diamond-Blackfan anaemia show abnormal expression of genes involved in protein synthesis, amino acid metabolism and cancer
Source: BMC Genomics. 2009 Sep 18;10:442. doi: 10.1186/1471-2164-10-442 (PMC2760583; doi:10.1186/1471-2164-10-442)
Supplement: Additional file 1 — Genes differentially expressed in DBA patients identified by microarray profiling. The table reports the probeset IDs which are differentially expressed in DBA patients relative to controls, with an FDR of 3%. The gene annotation, chromosome location and fold change of expression in patients relative to controls is also reported. [file 1471-2164-10-442-S1.pdf]

Additional file 1: Genes differentially expressed in DBA patients identified by microarray profiling (FDR 3%).

ProbesetID	Annotation	Gene	Chromosome	Fold change
204584_at	L1 cell adhesion molecule	L1CAM	X	38,445534
204614_at	serpin peptidase inhibitor, clade B (ovalbumin), member 2	SERPINB2	18	31,231388
202643_s_at	tumor necrosis factor, alpha-induced protein 3	TNFAIP3	6	21,932028
200953_s_at	cyclin D2	CCND2	12	20,61441
207386_at	cytochrome P450, family 7, subfamily B, polypeptide 1	CYP7B1	8	15,562447
204224_s_at	GTP cyclohydrolase 1 (dopa-responsive dystonia)	GCH1	14	15,506951
202644_s_at	tumor necrosis factor, alpha-induced protein 3	TNFAIP3	6	13,809285
207038_at	solute carrier family 16, member 6 (monocarboxylic acid transporter 7)	SLC16A6	17	13,714543
205266_at	leukemia inhibitory factor (cholinergic differentiation factor)	LIF	22	12,870623
210196_s_at	pregnancy specific beta-1-glycoprotein 1	PSG1	19	11,281122
209167_at	glycoprotein M6B	GPM6B	X	10,864785
210319_x_at	msh homeobox 2	MSX2	5	10,588639
213197_at	astrotactin 1	ASTN1	1	8,897583
217525_at	olfactomedin-like 1	OLFML1	11	7,9424553
203828_s_at	interleukin 32	IL32	16	7,8493257
210195_s_at	pregnancy specific beta-1-glycoprotein 1	PSG1	19	7,7765326
202158_s_at	CUG triplet repeat, RNA binding protein 2	CUGBP2	10	7,354651
213415_at	chloride intracellular channel 2	CLIC2	X	7,010556
201502_s_at	nuclear factor of kappa light polypeptide gene enhancer in B-cells inhibitor, alpha	NFKBIA	14	6,7364993
202638_s_at	intercellular adhesion molecule 1 (CD54), human rhinovirus receptor	ICAM1	19	6,531902
202156_s_at	CUG triplet repeat, RNA binding protein 2	CUGBP2	10	6,528866
203504_s_at	ATP-binding cassette, sub-family A (ABC1), member 1	ABCA1	9	6,348071
221523_s_at	Ras-related GTP binding D	RRAGD	6	6,0940895
218810_at	zinc finger CCCH-type containing 12A	ZC3H12A	1	6,076493
210735_s_at	carbonic anhydrase XII	CA12	15	5,7134695
204475_at	matrix metallopeptidase 1 (interstitial collagenase)	MMP1	11	5,6604667
204802_at	Ras-related associated with diabetes	RRAD	16	5,563784
215223_s_at	superoxide dismutase 2, mitochondrial	SOD2	6	5,5184064
210538_s_at	baculoviral IAP repeat-containing 3	BIRC3	11	5,4886994
205794_s_at	neuro-oncological ventral antigen 1	NOVA1	14	5,455379
216841_s_at	superoxide dismutase 2, mitochondrial	SOD2	6	5,3199086
221009_s_at	angiopoietin-like 4	ANGPTL4	19	5,192902
204508_s_at	carbonic anhydrase XII	CA12	15	5,0758915
220975_s_at	C1q and tumor necrosis factor related protein 1	C1QTNF1	17	4,9690013

218559_s_at	v-maf musculoaponeurotic fibrosarcoma oncogene homolog B (avian)	MAFB	20	4,821545
204803_s_at	Ras-related associated with diabetes	RRAD	16	4,798646
211668_s_at	plasminogen activator, urokinase	PLAU	10	4,7327657
218995_s_at	endothelin 1	EDN1	6	4,6506453
202510_s_at	tumor necrosis factor, alpha-induced protein 2	TNFAIP2	14	4,642153
202637_s_at	intercellular adhesion molecule 1 (CD54), human rhinovirus receptor	ICAM1	19	4,595
213817_at	NA	NA	NA	4,5680637
202157_s_at	CUG triplet repeat, RNA binding protein 2	CUGBP2	10	4,4877687
211828_s_at	TRAF2 and NCK interacting kinase	TNIK	3	4,1742644
37170_at	BMP2 inducible kinase	BMP2K	4	4,125386
207992_s_at	adenosine monophosphate deaminase (isoform E)	AMPD3	11	4,0883
221524_s_at	Ras-related GTP binding D	RRAGD	6	4,069406
202902_s_at	cathepsin S	CTSS	1	4,055197
204879_at	podoplanin	PDPN	1	3,997399
202014_at	protein phosphatase 1, regulatory (inhibitor) subunit 15A	PPP1R15A	19	3,9177356
214321_at	nephroblastoma overexpressed gene	NOV	8	3,89727
209959_at	nuclear receptor subfamily 4, group A, member 3	NR4A3	9	3,882347
207233_s_at	microphthalmia-associated transcription factor	MITF	3	3,8692203
37028_at	protein phosphatase 1, regulatory (inhibitor) subunit 15A	PPP1R15A	19	3,8512928
202509_s_at	tumor necrosis factor, alpha-induced protein 2	TNFAIP2	14	3,8088372
204702_s_at	nuclear factor (erythroid-derived 2)-like 3	NFE2L3	7	3,7832856
213638_at	phosphatase and actin regulator 1	PHACTR1	6	3,681895
204966_at	brain-specific angiogenesis inhibitor 2	BAI2	1	3,6686447
204760_s_at	nuclear receptor subfamily 1, group D, member 1	NR1D1	17	3,5897806
207978_s_at	nuclear receptor subfamily 4, group A, member 3	NR4A3	9	3,5630114
203823_at	regulator of G-protein signalling 3	RGS3	9	3,5363216
211823_s_at	paxillin	PXN	12	3,5036764
203505_at	ATP-binding cassette, sub-family A (ABC1), member 1	ABCA1	9	3,4963646
202890_at	microtubule-associated protein 7	MAP7	6	3,4433057
213038_at	IBR domain containing 3	IBRDC3	1	3,4396498
204472_at	GTP binding protein overexpressed in skeletal muscle	GEM	8	3,4271493
200935_at	calreticulin	CALR	19	3,4235466
210317_s_at	tyrosine 3-monooxygenase/tryptophan 5-monooxygenase activation protein, epsilon polypeptide	YWHAE	17	3,4218214
210692_s_at	solute carrier family 43, member 3	SLC43A3	11	3,4205127
205555_s_at	msh homeobox 2	MSX2	5	3,3823848
214797_s_at	PCTAIRE protein kinase 3	PCTK3	1	3,3810525
201473_at	jun B proto-oncogene	JUNB	19	3,3433392

203414_at	monocyte to macrophage differentiation-associated	MMD	17	3,331273
213988_s_at	spermidine/spermine N1-acetyltransferase 1	SAT1	X	3,2212915
205290_s_at	bone morphogenetic protein 2	BMP2	20	3,1958265
215485_s_at	intercellular adhesion molecule 1 (CD54), human rhinovirus receptor	ICAM1	19	3,158868
160020_at	matrix metallopeptidase 14 (membrane-inserted)	MMP14	14	3,0947516
218834_s_at	transmembrane protein 132A	TMEM132A	11	3,0584779
201490_s_at	peptidylprolyl isomerase F (cyclophilin F)	PPIF	10	3,0534277
210285_x_at	Wilms tumor 1 associated protein	WTAP	6	3,0376318
202393_s_at	Kruppel-like factor 10	KLF10	8	2,9982615
201489_at	peptidylprolyl isomerase F (cyclophilin F)	PPIF	10	2,962549
217279_x_at	matrix metallopeptidase 14 (membrane-inserted)	MMP14	14	2,9386556
207836_s_at	RNA binding protein with multiple splicing	RBPMS	8	2,9307668
220032_at	hypothetical protein FLJ21986	FLJ21986	7	2,8924313
203455_s_at	spermidine/spermine N1-acetyltransferase 1	SAT1	X	2,8907537
213107_at	TRAF2 and NCK interacting kinase	TNIK	3	2,8649378
204671_s_at	ankyrin repeat domain 6	ANKRD6	6	2,8585422
209928_s_at	musculin (activated B-cell factor-1)	MSC	8	2,821873
204132_s_at	forkhead box O3A	FOXO3A	6	2,8030708
40489_at	atrophin 1	ATN1	12	2,7663867
211596_s_at	leucine-rich repeats and immunoglobulin-like domains 1	LRIG1	3	2,7268617
210592_s_at	spermidine/spermine N1-acetyltransferase 1	SAT1	X	2,7085524
221484_at	UDP-Gal:betaGlcNAc beta 1,4- galactosyltransferase, polypeptide 5	B4GALT5	20	2,6985831
201236_s_at	BTG family, member 2	BTG2	1	2,6721601
220615_s_at	male sterility domain containing 1	MLSTD1	12	2,6445105
205205_at	v-rel reticuloendotheliosis viral oncogene homolog B, nuclear factor of kappa light polypeptide gene enhancer in B-cells 3 (avian)	RELB	19	2,639423
203434_s_at	membrane metallo-endopeptidase	MME	3	2,6378982
207425_s_at	septin 9	SEPT9	17	2,5923705
221752_at	slingshot homolog 1 (Drosophila)	SSH1	12	2,5756373
212090_at	glutamate receptor, ionotropic, N-methyl D-asparate-associated protein 1 (glutamate binding)	GRINA	8	2,5560393
209491_s_at	adenosine monophosphate deaminase (isoform E)	AMPD3	11	2,5372443
205479_s_at	plasminogen activator, urokinase	PLAU	10	2,5366268
201801_s_at	solute carrier family 29 (nucleoside transporters), member 1	SLC29A1	6	2,529054
209122_at	adipose differentiation-related protein	ADFP	9	2,5066714
209140_x_at	major histocompatibility complex, class I, B	HLA-B	6	2,502699
219675_s_at	UDP-glucuronate decarboxylase 1	UXS1	2	2,4718654
221727_at	SUB1 homolog (S. cerevisiae)	SUB1	5	2,4604828
202828_s_at	matrix metallopeptidase 14 (membrane-inserted)	MMP14	14	2,4468296

214716_at	BMP2 inducible kinase	BMP2K	4	2,427688
220941_s_at	chromosome 21 open reading frame 91	C21orf91	21	2,4060566
218066_at	solute carrier family 12 (potassium/chloride transporters), member 7	SLC12A7	5	2,3988705
208461_at	hypermethylated in cancer 1	HIC1	17	2,3863966
218880_at	FOS-like antigen 2	FOSL2	2	2,3810716
218294_s_at	nucleoporin 50kDa	NUP50	22	2,376102
207785_s_at	recombination signal binding protein for immunoglobulin kappa J region	RBPJ	4	2,3737972
218892_at	dachsous 1 (Drosophila)	DCHS1	11	2,3674352
207230_at	Cdon homolog (mouse)	CDON	11	2,338762
220603_s_at	multiple C2 domains, transmembrane 2	MCTP2	15	2,3367558
206636_at	RAS p21 protein activator 2	RASA2	3	2,3366497
208567_s_at	potassium inwardly-rectifying channel, subfamily J, member 12	KCNJ12	17	2,3076563
212956_at	TBC1 domain family, member 9 (with GRAM domain)	TBC1D9	4	2,3063335
205596_s_at	SMAD specific E3 ubiquitin protein ligase 2	SMURF2	17	2,281701
210655_s_at	forkhead box O3A	FOXO3A	6	2,2775753
203097_s_at	Rap guanine nucleotide exchange factor (GEF) 2	RAPGEF2	4	2,260679
203137_at	Wilms tumor 1 associated protein	WTAP	6	2,2470098
87100_at	abhydrolase domain containing 2	ABHD2	15	2,2420826
208685_x_at	bromodomain containing 2	BRD2	6	2,2417202
205174_s_at	glutaminyl-peptide cyclotransferase (glutaminyl cyclase)	QPCT	2	2,2239144
203927_at	nuclear factor of kappa light polypeptide gene enhancer in B-cells inhibitor, epsilon	NFKBIE	6	2,219177
212445_s_at	neural precursor cell expressed, developmentally down-regulated 4-like	NEDD4L	18	2,2023911
203795_s_at	B-cell CLL/lymphoma 7A	BCL7A	12	2,195319
204512_at	human immunodeficiency virus type I enhancer binding protein 1	HIVEP1	6	2,1923256
201703_s_at	protein phosphatase 1, regulatory (inhibitor) subunit 10	PPP1R10	6	2,1884
220034_at	interleukin-1 receptor-associated kinase 3	IRAK3	12	2,187441
210649_s_at	AT rich interactive domain 1A (SWI-like)	ARID1A	1	2,1632202
218733_at	male-specific lethal 2-like 1 (Drosophila)	MSL2L1	3	2,1426075
211141_s_at	CCR4-NOT transcription complex, subunit 3	CNOT3	19	2,1380587
209550_at	necdin homolog (mouse)	NDN	15	2,1337023
201206_s_at	ribosome binding protein 1 homolog 180kDa (dog)	RRBP1	20	2,1322062
208372_s_at	LIM domain kinase 1	LIMK1	7	2,1297653
206132_at	mutated in colorectal cancers	MCC	5	2,118436
209038_s_at	EH-domain containing 1	EHD1	11	2,1033323
219500_at	cardiotrophin-like cytokine factor 1	CLCF1	11	2,1023688
201393_s_at	insulin-like growth factor 2 receptor	IGF2R	6	2,0972528
214911_s_at	bromodomain containing 2	BRD2	6	2,0940754

203796_s_at	B-cell CLL/lymphoma 7A	BCL7A	12	2,0906243
204986_s_at	TAO kinase 2	TAOK2	16	2,0320442
203423_at	retinol binding protein 1, cellular	RBP1	3	2,0295143
203123_s_at	solute carrier family 11 (proton-coupled divalent metal ion transporters), member 2	SLC11A2	12	2,016867
209653_at	karyopherin alpha 4 (importin alpha 3)	KPNA4	3	2,0148556
201924_at	AF4/FMR2 family, member 1	AFF1	4	2,00754
209711_at	solute carrier family 35 (UDP-glucuronic acid/UDP-N-acetylgalactosamine dual transporter), member D1	SLC35D1	1	1,9932753
213295_at	cylindromatosis (turban tumor syndrome)	CYLD	16	1,9893612
212339_at	erythrocyte membrane protein band 4.1-like 1	EPB41L1	20	1,982505
217991_x_at	single stranded DNA binding protein 3	SSBP3	1	1,9798837
208420_x_at	suppressor of Ty 6 homolog (S. cerevisiae)	SUPT6H	17	1,9659269
213324_at	v-src sarcoma (Schmidt-Ruppin A-2) viral oncogene homolog (avian)	SRC	20	1,9570026
208290_s_at	eukaryotic translation initiation factor 5	EIF5	14	1,947333
209712_at	solute carrier family 35 (UDP-glucuronic acid/UDP-N-acetylgalactosamine dual transporter), member D1	SLC35D1	1	1,945003
221962_s_at	ubiquitin-conjugating enzyme E2H (UBC8 homolog, yeast)	UBE2H	7	1,9380258
209207_s_at	SEC22 vesicle trafficking protein homolog B (S. cerevisiae)	SEC22B	1	1,9038467
208706_s_at	eukaryotic translation initiation factor 5	EIF5	14	1,8846717
208831_x_at	suppressor of Ty 6 homolog (S. cerevisiae)	SUPT6H	17	1,8798479
201204_s_at	ribosome binding protein 1 homolog 180kDa (dog)	RRBP1	20	1,8755372
200916_at	transgelin 2	TAGLN2	1	1,8743255
212960_at	TBC1 domain family, member 9 (with GRAM domain)	TBC1D9	4	1,8742204
201702_s_at	protein phosphatase 1, regulatory (inhibitor) subunit 10	PPP1R10	6	1,866282
212135_s_at	ATPase, Ca++ transporting, plasma membrane 4	ATP2B4	1	1,8543861
216234_s_at	protein kinase, cAMP-dependent, catalytic, alpha	PRKACA	19	1,8478032
209636_at	nuclear factor of kappa light polypeptide gene enhancer in B-cells 2 (p49/p100)	NFKB2	10	1,84366
209324_s_at	regulator of G-protein signalling 16	RGS16	1	1,8392086
201995_at	exostoses (multiple) 1	EXT1	8	1,8353658
205548_s_at	BTG family, member 3	BTG3	21	1,8321908
213668_s_at	SRY (sex determining region Y)-box 4	SOX4	6	1,8313558
208812_x_at	major histocompatibility complex, class I, C	HLA-C	6	1,8218514
215050_x_at	mitogen-activated protein kinase-activated protein kinase 2	MAPKAPK2	1	1,8060554
214793_at	dual specificity phosphatase 7	DUSP7	3	1,80485
207740_s_at	nucleoporin 62kDa	NUP62	19	1,7953115
201794_s_at	Smg-7 homolog, nonsense mediated mRNA decay factor (C. elegans)	SMG7	1	1,7871369
213376_at	zinc finger and BTB domain containing 1	ZBTB1	14	1,7862701
209272_at	NGFI-A binding protein 1 (EGR1 binding protein 1)	NAB1	2	1,7713274

201585_s_at	splicing factor proline/glutamine-rich (polypyrimidine tract binding protein associated)	SFPQ	1	1,7428185
63825_at	abhydrolase domain containing 2	ABHD2	15	1,7367792
215660_s_at	microtubule associated serine/threonine kinase 2	MAST2	1	1,7287375
210269_s_at	chromosome X and Y open reading frame 3	CXYorf3	X	1,7200652
219397_at	coenzyme Q10 homolog B (S. cerevisiae)	COQ10B	2	1,7195199
215471_s_at	microtubule-associated protein 7	MAP7	6	1,7181154
212501_at	CCAAT/enhancer binding protein (C/EBP), beta	CEBPB	20	1,7026083
221903_s_at	cylindromatosis (turban tumor syndrome)	CYLD	16	1,6954912
208686_s_at	bromodomain containing 2	BRD2	6	1,6861874
211535_s_at	fibroblast growth factor receptor 1 (fms-related tyrosine kinase 2, Pfeiffer syndrome)	FGFR1	8	1,6831309
211182_x_at	runt-related transcription factor 1 (acute myeloid leukemia 1; aml1 oncogene)	RUNX1	21	1,6783204
214119_s_at	FK506 binding protein 1A, 12kDa	FKBP1A	20	1,6717649
203322_at	zinc finger protein 508	ZNF508	18	1,6628526
39582_at	cylindromatosis (turban tumor syndrome)	CYLD	16	1,6558889
204618_s_at	GA binding protein transcription factor, beta subunit 2	GABPB2	15	1,6356348
204433_s_at	spermatogenesis associated 2	SPATA2	20	1,6286346
204346_s_at	Ras association (RalGDS/AF-6) domain family 1	RASSF1	3	1,6036333
204096_s_at	elongation factor RNA polymerase II	ELL	19	1,5930148
217893_s_at	chromosome 1 open reading frame 108	C1orf108	1	1,5802705
209239_at	nuclear factor of kappa light polypeptide gene enhancer in B-cells 1 (p105)	NFKB1	4	1,5791218
210380_s_at	calcium channel, voltage-dependent, T type, alpha 1G subunit	CACNA1G	17	1,5768127
211743_s_at	proteoglycan 2, bone marrow (natural killer cell activator, eosinophil granule major basic protein)	PRG2	11	1,5641339
209834_at	carbohydrate (chondroitin 6) sulfotransferase 3	CHST3	10	1,5639827
212532_s_at	LSM12 homolog (S. cerevisiae)	LSM12	17	1,5601425
218312_s_at	zinc finger and SCAN domain containing 18	ZSCAN18	19	1,552449
220147_s_at	family with sequence similarity 60, member A	FAM60A	12	1,546936
213435_at	SATB homeobox 2	SATB2	2	1,5442784
217866_at	pre-mRNA cleavage factor I, 59 kDa subunit	FLJ12529	11	1,5378895
203183_s_at	SWI/SNF related, matrix associated, actin dependent regulator of chromatin, subfamily d, member 1	SMARCD1	12	1,5295463
208953_at	La ribonucleoprotein domain family, member 5	LARP5	10	1,5289757
212585_at	oxysterol binding protein-like 8	OSBPL8	12	1,5118544
49049_at	deltex 3 homolog (Drosophila)	DTX3	12	1,5060908
201698_s_at	splicing factor, arginine/serine-rich 9	SFRS9	12	1,4461972
208897_s_at	DEAD (Asp-Glu-Ala-Asp) box polypeptide 18	DDX18	2	1,4110554
203034_s_at	ribosomal protein L27a	RPL27A	11	0,8345711
200933_x_at	ribosomal protein S4, X-linked	RPS4X	X	0,787239
200053_at	sperm associated antigen 7	SPAG7	17	0,7744807

222229_x_at	hCG26523	hCG_26523	12	0,7723152
210775_x_at	caspase 9, apoptosis-related cysteine peptidase	CASP9	1	0,7722439
200038_s_at	ribosomal protein L17	RPL17	18	0,7630619
213377_x_at	similar to ribosomal protein S12	LOC440055	11	0,7627065
218654_s_at	mitochondrial ribosomal protein S33	MRPS33	7	0,7568266
211609_x_at	proteasome (prosome, macropain) 26S subunit, non-ATPase, 4	PSMD4	1	0,7482205
212039_x_at	ribosomal protein L3	RPL3	22	0,74541336
205162_at	excision repair cross-complementing rodent repair deficiency, complementation group 8	ERCC8	5	0,7427963
213347_x_at	ribosomal protein S4, X-linked	RPS4X	X	0,7351873
215582_x_at	MCM3 minichromosome maintenance deficient 3 (S. cerevisiae) associated protein	MCM3AP	21	0,7328866
200781_s_at	ribosomal protein S15a	RPS15A	16	0,72949064
214394_x_at	eukaryotic translation elongation factor 1 delta (guanine nucleotide exchange protein)	EEF1D	8	0,72586524
200090_at	farnesyltransferase, CAAX box, alpha	FNTA	8	0,7249798
45687_at	proline rich 14	PRR14	16	0,7243232
213969_x_at	ribosomal protein L29	RPL29	3	0,7208961
200809_x_at	ribosomal protein L12	RPL12	9	0,7203099
217256_x_at	NA	NA	NA	0,7168627
216274_s_at	SEC11 homolog A (S. cerevisiae)	SEC11A	15	0,716217
201572_x_at	dCMP deaminase	DCTD	4	0,7114956
203391_at	FK506 binding protein 2, 13kDa	FKBP2	11	0,71072316
200003_s_at	ribosomal protein L28	RPL28	19	0,7103508
200022_at	ribosomal protein L18	RPL18	19	0,71001697
214779_s_at	RUN and TBC1 domain containing 3	RUTBC3	22	0,7099239
217823_s_at	ubiquitin-conjugating enzyme E2, J1 (UBC6 homolog, yeast)	UBE2J1	6	0,7084667
214800_x_at	basic transcription factor 3	BTF3	5	0,7083612
214356_s_at	KIAA0368	KIAA0368	9	0,7082727
200932_s_at	dynactin 2 (p50)	DCTN2	12	0,70813656
210137_s_at	dCMP deaminase	DCTD	4	0,707879
204528_s_at	nucleosome assembly protein 1-like 1	NAP1L1	12	0,7068338
215160_x_at	similar to FRG1 protein (FSHD region gene 1 protein)	LOC642236	2	0,7057245
205682_x_at	apolipoprotein M	APOM	6	0,7051531
205760_s_at	8-oxoguanine DNA glycosylase	OGG1	3	0,70345485
46256_at	splA/ryanodine receptor domain and SOCS box containing 3	SPSB3	16	0,70314574
208692_at	ribosomal protein S3	RPS3	11	0,7030904
212270_x_at	ribosomal protein L17	RPL17	18	0,7007742
213031_s_at	WD repeat domain 73	WDR73	15	0,70067275
207573_x_at	ATP synthase, H+ transporting, mitochondrial F0 complex, subunit G	ATP5L	11	0,6978828

218667_at	praja 1	PJA1	X	0,69512016
208746_x_at	ATP synthase, H+ transporting, mitochondrial F0 complex, subunit G	ATP5L	11	0,6930288
200026_at	ribosomal protein L34	RPL34	4	0,6922072
213941_x_at	ribosomal protein S7	RPS7	2	0,69010276
210453_x_at	ATP synthase, H+ transporting, mitochondrial F0 complex, subunit G	ATP5L	11	0,6893073
200869_at	ribosomal protein L18a	RPL18A	19	0,6883658
212191_x_at	similar to ribosomal protein L13	LOC388344	17	0,68831307
214167_s_at	similar to ribosomal protein P0	RPLP0-like	2	0,6874756
212433_x_at	ribosomal protein S2	RPS2	16	0,68539345
216421_at	NA	NA	NA	0,6828673
209320_at	adenylate cyclase 3	ADCY3	2	0,6817202
212537_x_at	ribosomal protein L17	RPL17	18	0,67998016
207438_s_at	snurportin 1	SNUPN	15	0,6792787
200903_s_at	S-adenosylhomocysteine hydrolase	AHCY	20	0,67804605
201592_at	eukaryotic translation initiation factor 3, subunit 3 gamma, 40kDa	EIF3S3	8	0,67794013
213616_at	chromosome 18 open reading frame 10	C18orf10	18	0,67682815
214527_s_at	polyglutamine binding protein 1	PQBP1	X	0,6753828
203468_at	cyclin-dependent kinase (CDC2-like) 10	CDK10	16	0,6738647
222279_at	hypothetical protein FLJ35429	RP3-377H14.5	6	0,6718892
217780_at	chromosome 19 open reading frame 56	C19orf56	19	0,6717741
217776_at	retinol dehydrogenase 11 (all-trans/9-cis/11-cis)	RDH11	14	0,6694585
207335_x_at	ATP synthase, H+ transporting, mitochondrial F0 complex, subunit E	ATP5I	4	0,6689517
202484_s_at	methyl-CpG binding domain protein 2	MBD2	18	0,66793305
218845_at	dual specificity phosphatase 22	DUSP22	6	0,66763455
220014_at	proline rich 16	PRR16	5	0,66729647
202321_at	geranylgeranyl diphosphate synthase 1	GGPS1	1	0,66673875
201571_s_at	dCMP deaminase	DCTD	4	0,6636877
206169_x_at	zinc finger CCCH-type containing 7B	ZC3H7B	22	0,66121197
219590_x_at	DPH5 homolog (S. cerevisiae)	DPH5	1	0,65931606
203956_at	MORC family CW-type zinc finger 2	MORC2	22	0,6593057
209865_at	solute carrier family 35 (UDP-N-acetylglucosamine (UDP-GlcNAc) transporter), member A3	SLC35A3	1	0,6557375
219603_s_at	zinc finger protein 226	ZNF226	19	0,65500915
200715_x_at	ribosomal protein L13a	RPL13A	19	0,64997894
200823_x_at	ribosomal protein L29	RPL29	3	0,64815927
204387_x_at	mitochondrial ribosomal protein 63	MRP63	13	0,6475077
217737_x_at	chromosome 20 open reading frame 43	C20orf43	20	0,6471783
216620_s_at	Rho guanine nucleotide exchange factor (GEF) 10	ARHGEF10	8	0,6466012

212355_at	KIAA0323	KIAA0323	14	0,6455202
201781_s_at	aryl hydrocarbon receptor interacting protein	AIP	11	0,6445701
203327_at	insulin-degrading enzyme	IDE	10	0,6421286
219418_at	nonhomologous end-joining factor 1	NHEJ1	2	0,64204675
203478_at	NADH dehydrogenase (ubiquinone) 1, subcomplex unknown, 1, 6kDa	NDUFC1	4	0,6372388
220007_at	methyltransferase like 8	METTL8	2	0,63700676
221192_x_at	major facilitator superfamily domain containing 11	MFSD11	17	0,63297635
202184_s_at	nucleoporin 133kDa	NUP133	1	0,6328244
214075_at	neuron derived neurotrophic factor	NENF	1	0,6320581
216187_x_at	kinesin 2	KNS2	14	0,6317652
205105_at	mannosidase, alpha, class 2A, member 1	MAN2A1	5	0,6303134
207953_at	neuronal thread protein AD7c-NTP	AD7C-NTP	1	0,6278977
205854_at	tubby like protein 3	TULP3	12	0,62729764
216174_at	hepatocellular carcinoma-related HCRP1	HCRP1	6	0,6270118
202235_at	solute carrier family 16, member 1 (monocarboxylic acid transporter 1)	SLC16A1	1	0,62687993
201826_s_at	saccharopine dehydrogenase (putative)	SCCPDH	1	0,6255286
205743_at	SH3 and cysteine rich domain	STAC	3	0,6254721
213588_x_at	ribosomal protein L14	RPL14	3	0,6242107
204340_at	chromosome X open reading frame 12	CXorf12	X	0,6232359
201305_x_at	acidic (leucine-rich) nuclear phosphoprotein 32 family, member B	ANP32B	9	0,61963946
219336_s_at	activating signal cointegrator 1 complex subunit 1	ASCC1	10	0,6188982
215179_x_at	placental growth factor, vascular endothelial growth factor-related protein	PGF	14	0,61857915
216342_x_at	NA	NA	NA	0,61401135
205480_s_at	UDP-glucose pyrophosphorylase 2	UGP2	2	0,6122783
204800_s_at	dehydrogenase/reductase (SDR family) member 12	DHRS12	13	0,6107978
218709_s_at	intraflagellar transport 52 homolog (Chlamydomonas)	IFT52	20	0,6105939
219635_at	zinc finger protein 606	ZNF606	19	0,6101752
217719_at	eukaryotic translation initiation factor 3, subunit 6 interacting protein	EIF3S6IP	22	0,6100772
211114_x_at	survival of motor neuron protein interacting protein 1	SIP1	14	0,6081424
216570_x_at	similar to 60S ribosomal protein L29 (Cell surface heparin-binding protein HIP)	LOC283412	5	0,6067359
205573_s_at	sorting nexin 7	SNX7	1	0,6052896
221475_s_at	ribosomal protein L15	RPL15	3	0,6046415
201145_at	HCLS1 associated protein X-1	HAX1	1	0,6031907
201892_s_at	IMP (inosine monophosphate) dehydrogenase 2	IMPDH2	3	0,6025658
219979_s_at	chromosome 11 open reading frame 73	C11orf73	11	0,60206723
203232_s_at	ataxin 1	ATXN1	6	0,6018256
221589_s_at	aldehyde dehydrogenase 6 family, member A1	ALDH6A1	14	0,6015661

211623_s_at	fibrillarin	FBL	19	0,59925365
204929_s_at	vesicle-associated membrane protein 5 (myobrevin)	VAMP5	2	0,59884614
211115_x_at	survival of motor neuron protein interacting protein 1	SIP1	14	0,59774095
212530_at	NIMA (never in mitosis gene a)-related kinase 7	NEK7	1	0,5963266
219443_at	taspase, threonine aspartase, 1	TASP1	20	0,59492654
214902_x_at	LIM domain containing preferred translocation partner in lipoma	LPP	3	0,59397435
216383_at	hCG2040224	hCG_2040224	X	0,59369063
217797_at	ubiquitin-fold modifier conjugating enzyme 1	UFC1	1	0,5924007
220796_x_at	solute carrier family 35, member E1	SLC35E1	19	0,5923381
200036_s_at	ribosomal protein L10a	RPL10A	6	0,5916999
201258_at	ribosomal protein S16	RPS16	19	0,5904235
203551_s_at	COX11 homolog, cytochrome c oxidase assembly protein (yeast)	COX11	17	0,5896111
207769_s_at	polyglutamine binding protein 1	PQBP1	X	0,58904433
216806_at	NA	NA	NA	0,5890243
201268_at	non-metastatic cells 2, protein (NM23B) expressed in	NME2	17	0,58669263
222014_x_at	mitochondrial translation optimization 1 homolog (S. cerevisiae)	MTO1	6	0,5866419
219119_at	LSM8 homolog, U6 small nuclear RNA associated (S. cerevisiae)	LSM8	7	0,58587337
200705_s_at	eukaryotic translation elongation factor 1 beta 2	EEF1B2	2	0,5848407
205584_at	chromosome X open reading frame 45	CXorf45	X	0,58359325
219758_at	tetratricopeptide repeat domain 26	TTC26	7	0,582969
200847_s_at	transmembrane protein 66	TMEM66	8	0,5818581
209971_x_at	JTV1 gene	JTV1	7	0,5813371
218188_s_at	translocase of inner mitochondrial membrane 13 homolog (yeast)	TIMM13	19	0,57865506
219880_at	NA	NA	NA	0,57798153
214126_at	mitochondrial carrier triple repeat 1	MCART1	9	0,57712066
219122_s_at	tRNA-histidine guanylyltransferase 1-like (S. cerevisiae)	THG1L	5	0,5755856
213896_x_at	DnaJ (Hsp40) homolog, subfamily C, member 9	DNAJC9	10	0,57535523
217846_at	glutaminyl-tRNA synthetase	QARS	3	0,5752998
213675_at	NA	NA	NA	0,5742228
213940_s_at	formin binding protein 1	FNBP1	9	0,5741026
208726_s_at	eukaryotic translation initiation factor 2, subunit 2 beta, 38kDa	EIF2S2	20	0,5721113
213223_at	ribosomal protein L28	RPL28	19	0,5718402
202529_at	phosphoribosyl pyrophosphate synthetase-associated protein 1	PRPSAP1	17	0,5710564
210679_x_at	NA	NA	NA	0,56955004
221726_at	ribosomal protein L22	RPL22	1	0,56895936
217492_s_at	phosphatase and tensin homolog (mutated in multiple advanced cancers 1), pseudogene 1	PTENP1	9	0,56803095
213220_at	hypothetical protein LOC92482	LOC92482	10	0,5671628

210779_x_at	survival of motor neuron protein interacting protein 1	SIP1	14	0,563896
219348_at	uncharacterized hematopoietic stem/progenitor cells protein MDS032	MDS032	19	0,56352043
218225_at	ECSIT homolog (Drosophila)	ECSIT	19	0,5617446
201306_s_at	acidic (leucine-rich) nuclear phosphoprotein 32 family, member B	ANP32B	9	0,56119686
221706_s_at	uncharacterized hematopoietic stem/progenitor cells protein MDS032	MDS032	19	0,56109965
218258_at	polymerase (RNA) I polypeptide D, 16kDa	POLR1D	13	0,5591419
204290_s_at	aldehyde dehydrogenase 6 family, member A1	ALDH6A1	14	0,5577714
204053_x_at	phosphatase and tensin homolog (mutated in multiple advanced cancers 1)	PTEN	10	0,55744594
200843_s_at	glutamyl-prolyl-tRNA synthetase	EPRS	1	0,55728424
213485_s_at	ATP-binding cassette, sub-family C (CFTR/MRP), member 10	ABCC10	6	0,556776
217713_x_at	transmembrane protein 163	TMEM163	2	0,556009
216547_at	similar to laminin receptor 1 (ribosomal protein SA)	LOC127406	1	0,55597997
207730_x_at	hepatoma-derived growth factor-related protein 2	HDGF2	19	0,5555961
204332_s_at	aspartylglucosaminidase	AGA	4	0,55318576
204859_s_at	apoptotic peptidase activating factor 1	APAF1	12	0,5522114
65630_at	transmembrane protein 80	TMEM80	11	0,55154747
221935_s_at	chromosome 3 open reading frame 64	C3orf64	3	0,5493478
204554_at	protein phosphatase 1, regulatory (inhibitor) subunit 3D	PPP1R3D	20	0,54868424
207283_at	ribosomal protein L23a pseudogene 13	RPL23AP13	2	0,5486407
200802_at	seryl-tRNA synthetase	SARS	1	0,54696304
202098_s_at	protein arginine methyltransferase 2	PRMT2	21	0,54597276
217761_at	acireductone dioxygenase 1	ADI1	2	0,54418147
215204_at	SUMO1/sentrin specific peptidase 6	SENP6	6	0,54321694
218458_at	germ cell-less homolog 1 (Drosophila)	GMCL1	2	0,542698
216177_at	similar to 60S ribosomal protein L29 (P23)	LOC391132	1	0,5392261
213718_at	RNA binding motif protein 4	RBM4	11	0,53795075
209230_s_at	nuclear protein 1	NUPR1	16	0,5356156
221564_at	protein arginine methyltransferase 2	PRMT2	21	0,5311183
218440_at	methylcrotonoyl-Coenzyme A carboxylase 1 (alpha)	MCCC1	3	0,5310379
214605_x_at	G protein-coupled receptor 1	GPR1	2	0,5308611
214097_at	ribosomal protein S21	RPS21	20	0,5307938
220755_s_at	chromosome 6 open reading frame 48	C6orf48	6	0,5307007
218024_at	brain protein 44-like	BRP44L	6	0,53032064
213562_s_at	squalene epoxidase	SQLE	8	0,52793866
201922_at	TGF beta-inducible nuclear protein 1	TINP1	5	0,52519256
212732_at	maternally expressed 3	MEG3	14	0,52508515
208693_s_at	glycyl-tRNA synthetase	GARS	7	0,5186401

219076_s_at	peroxisomal membrane protein 2, 22kDa	PXMP2	12	0,5158136
209605_at	thiosulfate sulfurtransferase (rhodanese)	TST	22	0,51497024
219315_s_at	chromosome 16 open reading frame 30	C16orf30	16	0,5148234
211792_s_at	cyclin-dependent kinase inhibitor 2C (p18, inhibits CDK4)	CDKN2C	1	0,5120171
220720_x_at	family with sequence similarity 128, member B	FAM128B	2	0,5106839
221539_at	eukaryotic translation initiation factor 4E binding protein 1	EIF4EBP1	8	0,5105507
213693_s_at	mucin 1, cell surface associated	MUC1	1	0,5091612
217266_at	similar to ribosomal protein L15	tcag7.350	7	0,50678045
217810_x_at	leucyl-tRNA synthetase	LARS	5	0,501757
200629_at	tryptophanyl-tRNA synthetase	WARS	14	0,49850905
218553_s_at	potassium channel tetramerisation domain containing 15	KCTD15	19	0,4965397
212774_at	zinc finger protein 238	ZNF238	1	0,49369258
202609_at	epidermal growth factor receptor pathway substrate 8	EPS8	12	0,4932521
218716_x_at	mitochondrial translation optimization 1 homolog (S. cerevisiae)	MTO1	6	0,49276218
213238_at	ATPase, Class V, type 10D	ATP10D	4	0,49271503
221588_x_at	aldehyde dehydrogenase 6 family, member A1	ALDH6A1	14	0,4911269
206129_s_at	arylsulfatase B	ARSB	5	0,49026337
209759_s_at	dodecenoyl-Coenzyme A delta isomerase (3,2 trans-enoyl-Coenzyme A isomerase)	DCI	16	0,489561
205975_s_at	homeobox D1	HOXD1	2	0,47934923
202144_s_at	adenylosuccinate lyase	ADSL	22	0,4791538
215978_x_at	hypothetical protein LOC152719	LOC152719	4	0,4779303
212848_s_at	chromosome 9 open reading frame 3	C9orf3	9	0,47613284
200628_s_at	tryptophanyl-tRNA synthetase	WARS	14	0,4751582
202227_s_at	bromodomain containing 8	BRD8	5	0,47333884
206833_s_at	acylphosphatase 2, muscle type	ACYP2	2	0,47201437
214095_at	serine hydroxymethyltransferase 2 (mitochondrial)	SHMT2	12	0,4709392
215252_at	DnaJ (Hsp40) homolog, subfamily C, member 7	DNAJC7	17	0,47044224
200962_at	ribosomal protein L31	RPL31	2	0,4688049
218253_s_at	ligatin	LGTN	1	0,46486387
216147_at	septin 11	SEPT11	4	0,46461245
218237_s_at	solute carrier family 38, member 1	SLC38A1	12	0,46342477
210694_s_at	NA	NA	NA	0,46198815
217653_x_at	NA	NA	NA	0,45730305
206088_at	leucine rich repeat containing 37A	LRRC37A	17	0,4519046
217988_at	cyclin B1 interacting protein 1	CCNB1IP1	14	0,448093
201661_s_at	acyl-CoA synthetase long-chain family member 3	ACSL3	2	0,44647312
204333_s_at	aspartylglucosaminidase	AGA	4	0,44568515

212288_at	formin binding protein 1	FNBP1	9	0,44517416
205677_s_at	deleted in lymphocytic leukemia, 1	DLEU1	13	0,44435126
214096_s_at	serine hydroxymethyltransferase 2 (mitochondrial)	SHMT2	12	0,4430093
202847_at	phosphoenolpyruvate carboxykinase 2 (mitochondrial)	PCK2	14	0,44160336
65472_at	hypothetical LOC388969	LOC388969	2	0,43980938
219980_at	chromosome 4 open reading frame 29	C4orf29	4	0,43616793
211038_s_at	hypothetical protein MGC12760	MGC12760	1	0,43607572
205594_at	zinc finger protein 652	ZNF652	17	0,43309015
216064_s_at	aspartylglucosaminidase	AGA	4	0,43120405
215191_at	NA	NA	NA	0,43030414
217966_s_at	family with sequence similarity 129, member A	FAM129A	1	0,42454305
210701_at	craniofacial development protein 1	CFDP1	16	0,4212231
219366_at	apoptosis, caspase activation inhibitor	AVEN	15	0,40502974
217679_x_at	NA	NA	NA	0,3976473
210640_s_at	G protein-coupled receptor 30	GPR30	7	0,3940877
201505_at	laminin, beta 1	LAMB1	7	0,3856911
212811_x_at	solute carrier family 1 (glutamate/neutral amino acid transporter), member 4	SLC1A4	2	0,3842345
218517_at	PHD finger protein 17	PHF17	4	0,380069
203946_s_at	arginase, type II	ARG2	14	0,36954525
215641_at	SEC24 related gene family, member D (S. cerevisiae)	SEC24D	4	0,36869612
202363_at	sparc/osteonectin, cwcv and kazal-like domains proteoglycan (testican) 1	SPOCK1	5	0,3684402
215599_at	SMA4	SMA4	5	0,36161768
212810_s_at	solute carrier family 1 (glutamate/neutral amino acid transporter), member 4	SLC1A4	2	0,345768
205422_s_at	integrin, beta-like 1 (with EGF-like repeat domains)	ITGBL1	13	0,34050044
219773_at	NADPH oxidase 4	NOX4	11	0,33665383
215268_at	microtubule-actin crosslinking factor 1	MACF1	1	0,33569208
217127_at	cystathionase (cystathionine gamma-lyase)	CTH	1	0,32267526
219937_at	thyrotropin-releasing hormone degrading enzyme	TRHDE	12	0,31955618
217967_s_at	family with sequence similarity 129, member A	FAM129A	1	0,3138242
209610_s_at	solute carrier family 1 (glutamate/neutral amino acid transporter), member 4	SLC1A4	2	0,3108707
214927_at	integrin, beta-like 1 (with EGF-like repeat domains)	ITGBL1	13	0,3066272
213905_x_at	biglycan	BGN	X	0,30069315
201397_at	phosphoglycerate dehydrogenase	PHGDH	1	0,29107207
205893_at	neuroligin 1	NLGN1	3	0,28808743
201261_x_at	biglycan	BGN	X	0,2803747
216769_x_at	chromosome 9 open reading frame 150	C9orf150	9	0,28005874
210121_at	UDP-Gal:betaGlcNAc beta 1,3-galactosyltransferase, polypeptide 2	B3GALT2	1	0,26986724

210809_s_at	periostin, osteoblast specific factor	POSTN	13	0,2611895
221802_s_at	KIAA1598	KIAA1598	10	0,24443951
205047_s_at	asparagine synthetase	ASNS	7	0,24042608
205081_at	cysteine-rich protein 1 (intestinal)	CRIP1	14	0,23434593
217452_s_at	UDP-Gal:betaGlcNAc beta 1,3-galactosyltransferase, polypeptide 2	B3GALT2	1	0,22482036
220892_s_at	phosphoserine aminotransferase 1	PSAT1	9	0,21060894
212311_at	KIAA0746 protein	KIAA0746	4	0,1891195
205132_at	actin, alpha, cardiac muscle 1	ACTC1	15	0,15708469
207302_at	sarcoglycan, gamma (35kDa dystrophin-associated glycoprotein)	SGCG	13	0,12657614
212314_at	KIAA0746 protein	KIAA0746	4	0,11605084
206373_at	Zic family member 1 (odd-paired homolog, Drosophila)	ZIC1	3	0,09232327
219478_at	WAP four-disulfide core domain 1	WFDC1	16	0,0805757
205713_s_at	cartilage oligomeric matrix protein	COMP	19	0,06623136
