# Supplementary material for: Fibroblasts from patients with Diamond-Blackfan anaemia show abnormal expression of genes involved in protein synthesis, amino acid metabolism and cancer
Source: BMC Genomics. 2009 Sep 18;10:442. doi: 10.1186/1471-2164-10-442 (PMC2760583; doi:10.1186/1471-2164-10-442)
Supplement: Additional file 2 — Differentially expressed genes in females relative to males. The table reports the probeset IDs which are differentially expressed in females relative to males, with an FDR of 10%. The gene annotation, chromosome location and fold change of expression in females relative to males is also reported. [file 1471-2164-10-442-S2.pdf]

Additional file 2: Differentially expressed genes in females relative to males (FDR 10%).

ProbesetID	Annotation	Gene	Chromosome	Fold change
221728_x_at	X (inactive)-specific transcript	XIST	X	83,71634
214218_s_at	X (inactive)-specific transcript	XIST	X	48,653202
205249_at	early growth response 2 (Krox-20 homolog, Drosophila)	EGR2	10	6,687153
204830_x_at	pregnancy specific beta-1-glycoprotein 5	PSG5	19	4,378566
220049_s_at	programmed cell death 1 ligand 2	PDCD1LG2	9	3,6263728
201645_at	tenascin C (hexabrachion)	TNC	9	3,6162076
217635_s_at	polymerase (DNA directed), gamma	POLG	15	0,6805993
212498_at	NA	NA	NA	0,67220706
212251_at	metadherin	MTDH	8	0,66292065
203558_at	cullin 7	CUL7	6	0,6498589
209127_s_at	squamous cell carcinoma antigen recognized by T cells 3	SART3	12	0,64063835
211066_x_at	protocadherin gamma subfamily C, 3	PCDHGC3	5	0,6294207
208503_s_at	GATA zinc finger domain containing 1	GATAD1	7	0,60239685
221272_s_at	chromosome 1 open reading frame 21	C1orf21	1	0,5321209
205002_at	AT hook, DNA binding motif, containing 1	AHDC1	1	0,48217613
217550_at	activating transcription factor 6	ATF6	1	0,42879397
205197_s_at	ATPase, Cu++ transporting, alpha polypeptide (Menkes syndrome)	ATP7A	X	0,42660233
213274_s_at	cathepsin B	CTSB	8	0,42486367
204060_s_at	protein kinase, X-linked	PRKX	X	0,3382931
213252_at	SH3 and PX domains 2A	SH3PXD2A	10	0,3083455
211685_s_at	neurocalcin delta	NCALD	8	0,2830072
206876_at	single-minded homolog 1 (Drosophila)	SIM1	6	0,2747715
206279_at	protein kinase, Y-linked	PRKY	Y	0,24020328
207063_at	chromosome Y open reading frame 14	CYorf14	Y	0,22663628
204410_at	eukaryotic translation initiation factor 1A, Y-linked	EIF1AY	Y	0,16866268
214131_at	chromosome Y open reading frame 15B	CYorf15B	Y	0,1648593
205001_s_at	DEAD (Asp-Glu-Ala-Asp) box polypeptide 3, Y-linked	DDX3Y	Y	0,14647998
207703_at	neuroligin 4, Y-linked	NLGN4Y	Y	0,06424095
206624_at	ubiquitin specific peptidase 9, Y-linked (fat facets-like, Drosophila)	USP9Y	Y	0,058675706
204409_s_at	eukaryotic translation initiation factor 1A, Y-linked	EIF1AY	Y	0,05459688
206700_s_at	jumonji, AT rich interactive domain 1D	JARID1D	Y	0,054338217
205000_at	DEAD (Asp-Glu-Ala-Asp) box polypeptide 3, Y-linked	DDX3Y	Y	0,022558337
201909_at	ribosomal protein S4, Y-linked 1	RPS4Y1	Y	0,01256958
