# Supplementary material for: Fibroblasts from patients with Diamond-Blackfan anaemia show abnormal expression of genes involved in protein synthesis, amino acid metabolism and cancer
Source: BMC Genomics. 2009 Sep 18;10:442. doi: 10.1186/1471-2164-10-442 (PMC2760583; doi:10.1186/1471-2164-10-442)
Supplement: Additional file 3 — Differentially expressed genes in female patients relative to male patients. The table reports the probeset IDs which are differentially expressed in female DBA patients relative to male DBA patients, with an FDR of 10%. The gene annotation, chromosome location and fold change of expression in females relative to males is also reported. [file 1471-2164-10-442-S3.pdf]

Additional file 3: Differentially expressed genes in female patients relative to male patients (FDR 10%).

ProbesetID	Annotation	Gene	Chromosome	Fold change
221728_x_at	X (inactive)-specific transcript	XIST	X	83,52757
214218_s_at	X (inactive)-specific transcript	XIST	X	40,159718
205001_s_at	DEAD (Asp-Glu-Ala-Asp) box polypeptide 3, Y-linked	DDX3Y	Y	0,10990563
204409_s_at	eukaryotic translation initiation factor 1A, Y-linked	EIF1AY	Y	0,05324197
206700_s_at	jumonji, AT rich interactive domain 1D	JARID1D	Y	0,040198654
205000_at	DEAD (Asp-Glu-Ala-Asp) box polypeptide 3, Y-linked	DDX3Y	Y	0,022345949
201909_at	ribosomal protein S4, Y-linked 1	RPS4Y1	Y	0,015291744
