# Supplementary material for: Fibroblasts from patients with Diamond-Blackfan anaemia show abnormal expression of genes involved in protein synthesis, amino acid metabolism and cancer
Source: BMC Genomics. 2009 Sep 18;10:442. doi: 10.1186/1471-2164-10-442 (PMC2760583; doi:10.1186/1471-2164-10-442)
Supplement: Additional file 4 — Primers used in the expression analysis by qRT-PCR. The table reports the sequence of both forward and reverse primers used to amplify genes in the validation experiments by qRT-PCR. The final concentration used in the reactions is also indicated for each primer. [file 1471-2164-10-442-S4.pdf]

Additional file 4: Primers used in the expression analysis by qRT-PCR.

Gene	Primer	Sequence 5' - 3'	Concentration (nM)
ACTB	Forward	CGCCGCCAGCTCACCATG	250
	Reverse	CACGATGGAGGGGAAGACGG	250
AMPD3	Forward	TCCGAGTTCAAAGAGTTGAAGAGT	250
	Reverse	GCTCCGTCTGGTATGTGT	250
CCND2	Forward	TTGTGATGCCCTGACTGAGC	500
	Reverse	GTTGGTCCTGACGGTACGG	500
COMP	Forward	TGGCATCCAACTCAAGGCT	250
	Reverse	CCCTCATAGAATCGCACCCTG	250
TNFAIP3	Forward	TTGTGGCGCTGAAAACGAAC	250
	Reverse	GTGTCTGTTTCCTTGAGCGT	500
SOD2	Forward	CACTTACAAATTGCTGCTTGTCCA	500
	Reverse	TACTGAAGGTAGTAAGCGTGCTC	500
WARS	Forward	TCATATTCTCTGACCTGGACTACAT	500
	Reverse	CCGAAAATGCCTTTCACTTGG	500
ZIC1	Forward	ACAAAAGGACGCACACAGGG	500
	Reverse	GGATGCGTGTAGGACTTGTCG	500
